# Modulation of the central nervous system immune response and neuroinflammation via Wnt signaling in health and neurodegenerative diseases

**DOI:** 10.1002/ibra.12185

**Published:** 2024-12-02

**Authors:** Kevin Fang

**Affiliations:** ^1^ Living Systems Institute University Exeter Exeter UK

**Keywords:** astrocytes, microglia, neurodegenerative diseases, neuroinflammation, Wnt signaling

## Abstract

The immune response in the central nervous system (CNS) is a highly specialized and tightly regulated process essential for maintaining neural health and protecting against pathogens and injuries. The primary immune cells within the CNS include microglia, astrocytes, T cells, and B cells. They work together, continuously monitor the CNS environment for signs of infection, injury, or disease, and respond by phagocytosing debris, releasing cytokines, and recruiting other immune cells. In addition to providing neuroprotection, these immune responses must be carefully balanced to prevent excessive inflammation that can lead to neuronal damage and contribute to neurodegenerative diseases. Dysregulated immune responses in the CNS are implicated in various neurodegenerative diseases such as Alzheimer's disease, Parkinson's disease, and amyotrophic lateral sclerosis. Wnt signaling is a crucial pathway in the CNS that regulates various cellular processes critical for brain development, function, and maintenance. Despite enhancing immune responses in the health CNS, dysregulated Wnt signaling exacerbates neuroinflammation in the neurodegenerative brains. This review summarized the role of Wnt signaling in regulating immune response under different conditions. We then examined the role of immune response in healthy brains and during the development of neurodegenerative diseases. We also discussed therapeutic intervention in various neurodegenerative diseases through the modulation of the Wnt signaling pathway and neuroinflammation and highlighted challenges and limitations in current clinical trials.

## Graphical abstract

Wnt signaling regulates the immune response in the healthy central nervous system and the diseased brain. Fully regulated immune cells by Wnt signaling work together to provide neuroprotection and maintain homeostasis in the healthy brain, while dysregulated immune cells by disrupted Wnt signaling result in neurodegenerative diseases.

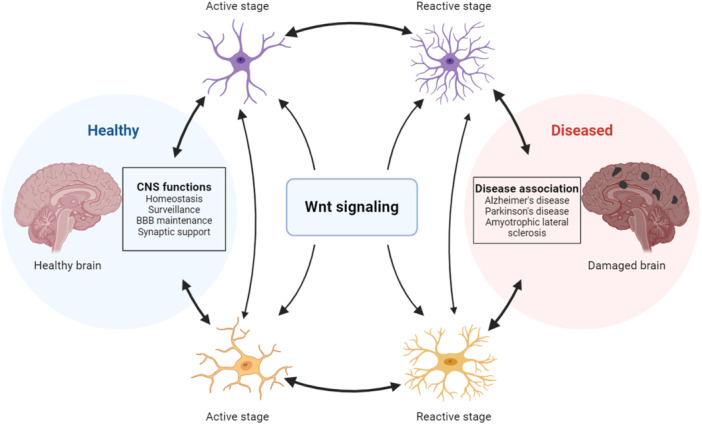

## 1 INTRODUCTION

Neurodegenerative diseases are a diverse group of disorders characterized by the progressive degeneration of the structure and function of the nervous system. These diseases, such as Alzheimer's disease (AD), Parkinson's disease (PD), and amyotrophic lateral sclerosis (ALS) predominantly affect neurons, the primary building blocks of the nervous system responsible for transmitting signals throughout the body. As neurons are typically incapable of regenerating, their loss leads to a gradual decline in cognitive, motor, and sensory functions. AD is a progressive neurodegenerative disorder and the most common cause of dementia, affecting millions of people worldwide. It is characterized by a gradual decline in cognitive functions, including memory, thinking, and reasoning skills, which significantly interfere with daily life and activities.^1^, ^2^ PD primarily affects movement control due to the loss of dopamine‐producing neurons in the substantia nigra, a region of the brain critical for coordinating movement.^2^ ALS, also known as Lou Gehrig's disease, is a progressive neurodegenerative disorder that primarily affects motor neurons in the brain and spinal cord.^3^ As ALS progresses, these neurons degenerate and die, leading to a gradual loss of muscle control. This results in muscle weakness, twitching, and eventually paralysis. Patients with ALS may initially experience symptoms like difficulty speaking, swallowing, or walking, which progressively worsen over time.^3^ The etiology of these neurodegenerative diseases is complex and multifactorial, involving a combination of genetic, environmental, and lifestyle factors. Common pathological features include abnormal protein aggregations, oxidative stress, mitochondrial dysfunction, and chronic neuroinflammation.^4^ For instance, AD is characterized by the accumulation of amyloid‐beta (Aβ) plaques and neurofibrillary tangles composed of hyperphosphorylated tau protein in the brain.^5^ These pathological hallmarks disrupt neuronal communication, lead to synaptic loss, and trigger widespread neurodegeneration. Aberrant expression of synuclein alpha is found in PD and it encodes alpha‐synuclein, a protein that aggregates to form Lewy bodies, a pathological hallmark of PD.^6^ ALS is characterized by the accumulation of misfolded proteins, such as aggregation of TAR DNA‐binding protein 43 (TDP‐43) and superoxide dismutase 1 (SOD1) which form toxic aggregates within motor neurons.^7^

In the brain, the immune response in the central nervous system (CNS) is essential for maintaining homeostasis, responding to injury, and protecting against infections. The immune cells of the CNS include microglia, astrocytes, T/B cells, and perivascular macrophages.^8^, ^9^ Among them, microglia are the main primary immune cells in the CNS, constantly surveying the microenvironment and responding to injury or infection with a range of activation states. These cells can adopt a neuroprotective phenotype by releasing cytokines and chemokines to recruit peripheral immune cells and clear pathogens. Astrocytes, another type of glial cell, maintain blood–brain barrier (BBB) integrity, regulate neurotransmitter levels, and respond to injury by becoming reactive, a process known as astrogliosis. Reactive astrocytes can produce inflammatory mediators and interact with other immune cells, modulating the inflammatory response. While in neurodegenerative diseases, the immune response in the CNS becomes a double‐edged sword. Despite initial protective function, chronic activation of immune cells and persistent inflammation can exacerbate neuronal damage and contribute to disease progression.^10^ Wnt signaling pathway is a highly conserved pathway involved in many aspects of development and plays a crucial role in regulating immune responses in the CNS. This pathway influences the proliferation, differentiation, and the behavior of immune cells.^11^ Normal Wnt signaling shows neuroprotective functions in the CNS by enhancing immune responses during injury and infection. However, during the development of neurodegenerative diseases, Wnt signaling is dysregulated, and this dysregulation further complicates these diseases by altering the balance of immune responses in the CNS. This review explored how Wnt signaling is involved in regulating in neuron immune system to affect neurodegenerative disease. Understanding the role of Wnt signaling in these immune responses opens new avenues for therapeutic interventions aimed at modulating this pathway to reduce neuroinflammation and neuronal damage in neurodegenerative diseases.

## 2 IMMUNE RESPONSE IN THE CNS AND NEURODEGENERATIVE DISEASES

### 2.1 Immune response in the healthy CNS

The immune response is a complex and finely regulated process crucial for maintaining homeostasis in the CNS. The immune cells include microglia, astrocytes, T cells, and B cells. They constantly monitor the CNS environment and can become activated in response to pathogens, injury, or disease, leading to the release of cytokines, chemokines, and other inflammatory mediators.

#### 2.1.1 Microglia

Microglial cells are the primary resident immune cells of the CNS. Originating from myeloid progenitors during embryogenesis, microglia are pivotal in maintaining CNS homeostasis, promoting neural circuit development, and modulating synaptic activity for neuronal communication.^12^, ^13^

**Figure 1:**
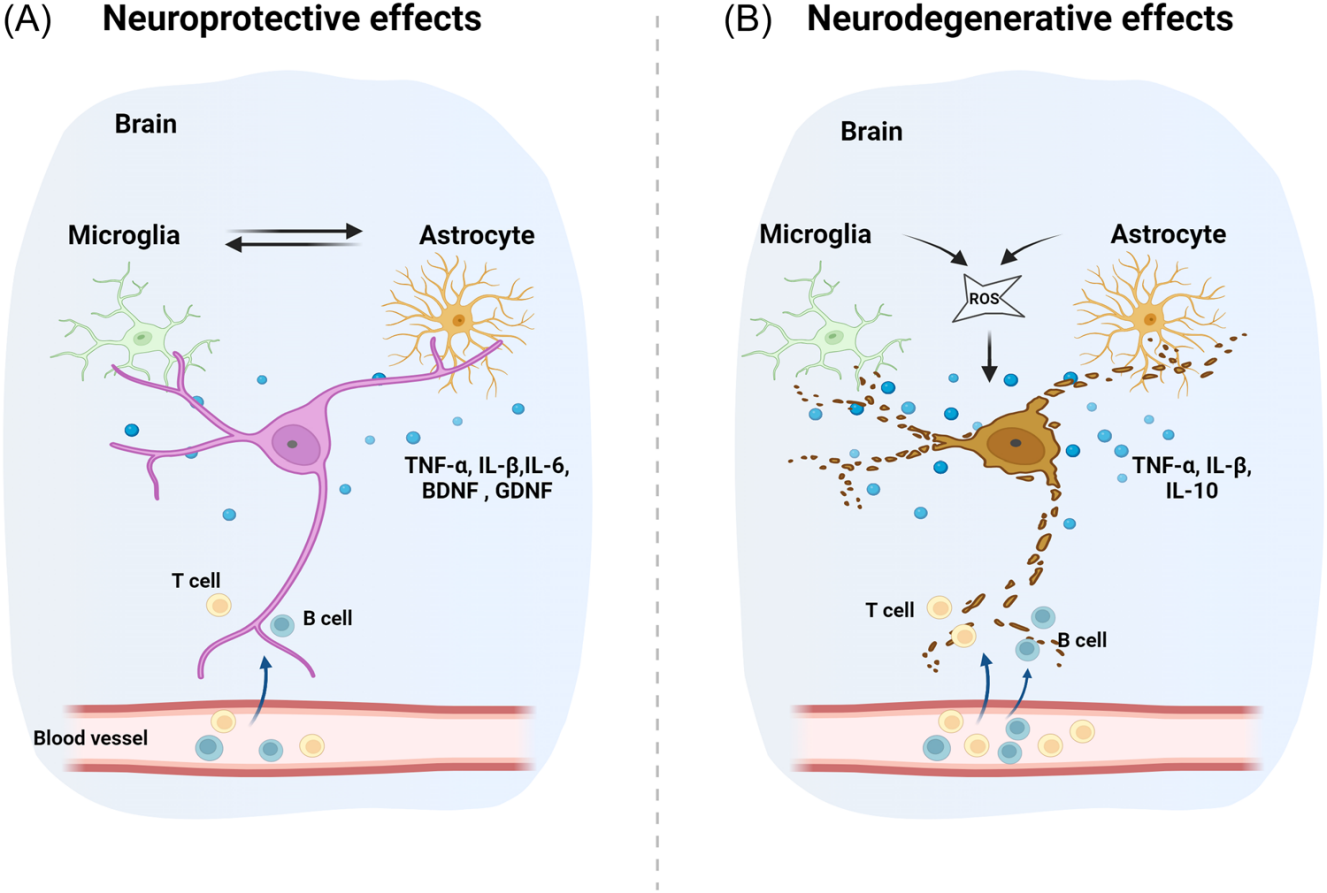
Immune responses in the healthy and neurodegeneration brain. (A) In the healthy brain, microglia and astrocytes produce cytokines (e.g., TNF‐α, IL‐β, IL‐6) in response to homeostatic unbalance and neurotrophic factors (e.g., BDNF, GDNF) to help neurogenesis. Peripheral immune cells, including T cells and B cells, migrate into the CNS to protect neurons. (B) In the neurodegeneration process, chronic immune responses and ROS initiated by microglia and astrocytes constantly produce cytokines and chemokines, which result in damage to neurons. More reactive T cells and B cells migrate into the CNS to kill neurons. IL‐6, interleukin‐6; IL‐β, interleukin‐β; BDNF, brain‐derived neurotrophic factor; CNS, central nervous system; GDNF, glial cell‐derived neurotrophic factor; ROS, reactive oxygen species; TNF‐α, tumor necrosis factor‐α. [Color figure can be viewed at wileyonlinelibrary.com]

Upon detecting disturbances in the CNS environment through pattern recognition receptors, including Toll‐like receptors and NOD‐like receptors on the membrane surface, microglial cells transformed into an active state.^14^, ^15^ Active microglia produce a wide array of cytokines and chemokines that mediate inflammatory responses, recruit peripheral immune cells, and influence the behavior of neurons and other glial cells. For instance, microglia produce proinflammatory cytokines such as tumor necrosis factor‐α (TNF‐α), interleukin‐1β (IL‐1β), and interleukin‐6 (IL‐6) to initiate and propagate inflammatory responses^16^, ^17^ (Figure 1A). Meanwhile, anti‐inflammatory cytokines like interleukin‐10 (IL‐10) and transforming growth factor beta (TGF‐β), produced by microglia, help resolve inflammation and promote tissue repair.^18^ Microglia attract peripheral immune cells to the sites of injury or infection to maintain homeostasis in the CNS by secreting chemokines, for instance, C‐C motif ligand 2 and C‐X‐C motif chemokine ligand 8.^19^, ^20^ Evidence also shows that purinergic receptors have their functions in activating microglia. For example, purinergic receptor P2Y12 detects adenosine triphosphate (ATP) released from damaged or stressed cells and facilitates rapid microglial chemotaxis toward the injury site.^21^ Phagocytosis is a fundamental function of microglia. Triggering receptor expressed on myeloid cells 2 (TREM2) is a transmembrane protein receptor that is expressed by microglia cells, and it is involved in the recognition and phagocytosis of apoptotic neurons.^22^ Moreover, microglia support neurogenesis in specific brain regions. For example, in the hippocampus, microglia release neurotrophic factors such as brain‐derived neurotrophic factor (BDNF) and nerve growth factor (NGF) to promote the proliferation and differentiation of neural progenitor cells.^23^, ^24^ Microglia also modulate synaptic activity by releasing insulin‐like growth factor 1.^25^ Moreover, following CNS injury, microglia contribute to repair mechanisms by releasing factors that promote the regeneration of neural tissue.^26^ They can form a glial scar to isolate the injured area and hinder regenerative processes. However, in various CNS diseases, dysregulated microglial activation is a common pathological feature. Persistent activation of microglia and the chronic production of proinflammatory cytokines can exacerbate neuronal damage and contribute to the progression of these diseases.^27^

#### 2.1.2 Astrocytes

Astrocytes, star‐shaped glial cells found throughout the brain and spinal cord, are the most abundant cell type in the CNS, and they play multifaceted roles crucial for the maintenance of CNS homeostasis, synaptic function and plasticity, BBB integrity, and the response to injury.

Same to microglia, as shown in Figure 1A, astrocytes exhibit inflammatory and immune responses by secreting cytokines and chemokines including IL‐6, TGF‐β.^28^, ^29^ Furthermore, astrocytes express glutamate transporters to modulate neurotransmitter homeostasis by transporting several neurotransmitters, including glutamate, ATP, and gamma‐aminobutyric acid.^30^, ^31^ Receptors glucose transporter and monocarboxylate transporter expressed by astrocytes can convert glucose from the bloodstream into lactate through glycolysis, providing an energy source for neurons.^32^ Astrocytes also support neuronal survival, growth, and regeneration by secreting neurotrophic factors. Astrocytes modulate the maintenance of the BBB, a selective barrier that controls the passage of substances from the bloodstream into the brain. They contribute to the integrity and function of the BBB by unsheathing blood vessels with their end feet, controlling the passage of substances from the bloodstream into brain.^33^ While reactive astrocytes can initially protect neurons, constantly activated astrocytes can exacerbate neuroinflammation and release proinflammatory cytokines and chemokines that cause damage to neurons. They also influence the activity of microglia, further contributing to the inflammatory environment and causing neurodegenerative diseases.^9^, ^34^

#### 2.1.3 Peripheral immune cell infiltration

Under certain conditions, such as infection, autoimmune disease, or injury, peripheral immune cells can cross the BBB. In Figure 1A, this process involves the migration of immune cells such as T cells, B cells, macrophages, and neutrophils from the peripheral circulation into the CNS, where they can exert both protective and detrimental effects.^35^ For instance, cytotoxic T cells (CD8+) can directly kill infected or damaged neurons by recognizing specific antigens.^36^ And B cells can secrete anti‐inflammatory cytokines like IL‐10, which suppress immune responses and promote immune tolerance.^37^ Macrophages produce proinflammatory or anti‐inflammatory cytokines, depending on their activation state (M1 or M2 polarization).^38^ Similarly, persistent activation of T cells, B cells, and macrophages can lead to chronic neuroinflammation, contributing to neuronal damage and disease progression in conditions like AD and PD.^39^

### 2.2 Immune response in neurodegenerative diseases

The immune response has shown its importance in maintaining homeostasis in the healthy CNS. However, abnormal immune response influences disease progression and severity during the development of degenerative diseases. Neurodegenerative diseases, including AD, PD, and ALS, are characterized by the progressive loss of structure or function of neurons. Although the immune system's role in these diseases is complex and not fully understood, it is clear that the involvement of both innate and adaptive immune responses within the CNS significantly impacts disease outcomes.

#### 2.2.1 Immune response in AD

AD is a chronic neurodegenerative disease that is the most common cause of dementia in older adults. It is characterized by progressive memory loss, cognitive decline, and behavioral changes. Increasing evidence suggests that the immune system plays a significant role in the pathogenesis and progression of AD. Primary immune cells of the CNS, including microglia and astrocytes, respond to AD's pathological hallmarks, Aβ plaques, and tau tangles. For example, a recent study shows that apolipoprotein E mediates microglial activation by impairing lipid metabolism and microglial responses to amyloid pathology in the AD mice model.^40^ The receptor TREM2 on microglia binds to Aβ to promote microglial activation and phagocytosis, and variants in TREM2 are associated with increased risk for AD.^41^, ^42^ Furthermore, overactivated immune response leads to neuroinflammation, which results in sustained release of proinflammatory cytokines, chemokines, and reactive oxygen species (ROS), contributing to a proinflammatory environment that can exacerbate neuronal damage (Figure 1B).^43^, ^44^, ^45^, ^46^, ^47^ For instance, the inflammatory cytokine IL‐1β secreted by microglia is upregulated in AD and is associated with decreases of synaptophysin, consequent synaptic dysfunction, and neuronal loss.^48^, ^49^ The nuclear factor‐kappaB (NF‐κB) pathway is the key signaling mechanism in response to cytokines, ROS, and other stress signal. Persistent activation of NF‐κB signaling is found in microglia, and astrocytes contribute to the chronic production of proinflammatory mediators in AD.^50^ Moreover, chronic inflammation can compromise the integrity of the BBB, facilitating the entry of peripheral immune cells into the brain and further contributing to neuroinflammation.^51^ Overall, the immune system plays a critical role in the pathogenesis and progression of AD. Neuroinflammation driven by microglial and astrocytic activation, peripheral immune cell infiltration, and the interplay between them contribute to neuronal damage and cognitive decline.

#### 2.2.2 Immune response in PD

PD is a progressive neurodegenerative disorder characterized primarily by motor symptoms such as tremors, rigidity, bradykinesia, and postural instability. Recent research has highlighted the necessity of the immune system in the pathogenesis and progression of PD, involving the activation of microglia, astrocytes, and the brain's resident immune cells. In PD, microglia become active in response to neuronal injury, alpha‐synuclein aggregation, and other stimuli. Active microglia exhibit a proinflammatory phenotype (M1), characterized by the production of cytokines (e.g., TNF‐α, IL‐1β, IL‐6), which can impair neuronal survual.^52^, ^53^ The anti‐inflammatory (M2) microglia, which help in tissue repair and resolution of inflammation, are significant but seem to be diminished in PD.^54^, ^55^ Astrocytes, when reactivated, release inflammatory mediators that contribute to the neuroinflammatory milieu. Additionally, T‐cell infiltration in the PD brain has been reported, suggesting a link between peripheral immune activation and neuroinflammation in PD.^56^ Several genes associated with PD were reported to influence immune function. For example, mutations in the leucine‐rich repeat kinase 2 (LRRK2) gene, expressed in immune cells and involved in the regulation of inflammation, are associated with both familial and sporadic PD.^57^ Similarly, polymorphisms in the major histocompatibility complex, Class II, DR Beta 1 gene, which is involved in antigen presentation, have been associated with an increased risk of PD.^58^, ^59^ All these studies suggest the necessity of the immune system in the pathogenesis of PD. Neuroinflammation driven by microglial activation and peripheral immune responses contribute to the disease's progression.

#### 2.2.3 Immune response in ALS

ALS is a progressive neurodegenerative disease characterized by the degeneration of motor neurons, leading to muscle weakness, atrophy, and, ultimately, respiratory failure. The exact etiology of ALS is not fully understood, but it involves a complex interplay of genetic, environmental, and molecular factors. More and more research studies have indicated the significance of the immune system in the pathogenesis and progression of ALS. The immune cells, like microglia and astrocytes, are activated in the CNS and contribute to the inflammatory environment surrounding motor neurons. For example, microglia produce anti‐inflammatory cytokines and neurotrophic factors to support motor neuron survival and tissue repair. However, during ALS development, microglia exhibit neurotoxic phenotypes by releasing proinflammatory cytokines (e.g., TNF‐α, IL‐1β, IL‐6), ROS, and nitric oxide, which contribute to motor neuron injury and death.^60^ In the meantime, astrocytes contribute to the immune inflammation in ALS by producing neurotoxic factors like glutamate. More peripheral immune cells infiltrate into the CNS in ALS.^61^ Both CD4+ and CD8 + T cells have been found in the spinal cords of ALS patients. CD8 + T cells can directly kill motor neurons, while CD4 + T cells can modulate the activity of other immune cells, including microglia and astrocytes.^62^ In summary, immune‐mediated inflammation is a significant contributor to the pathogenesis of ALS. The chronic activation of microglia and astrocytes, abnormal infiltration of peripheral immune cells, and production of proinflammatory mediators lead to a harmful environment for motor neurons.

## 3 WNT SIGNALING IN REGULATING THE CNS IMMUNE RESPONSE

In the past decades, research has demonstrated that the immune response in the CNS receives a great influence from Wnt signaling. Wnt signaling impacts both the innate and adaptive immune responses and is involved in the regulation of inflammation, immune cell trafficking, and the maintenance of the BBB.

### 3.1 Wnt signaling pathways

**Figure 2:**
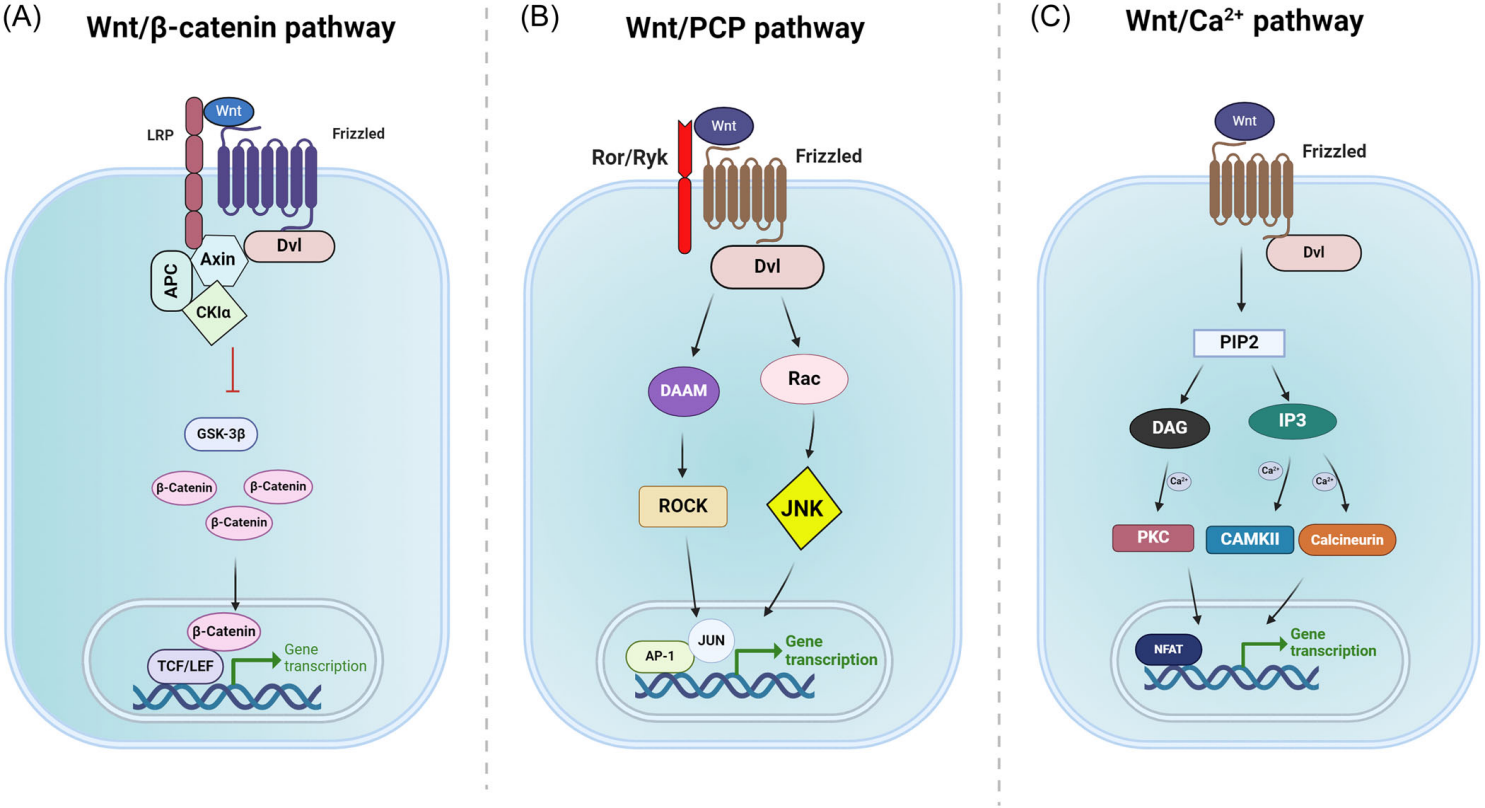
Wnt signaling pathways. (A) The Wnt/β‐catenin signaling pathway. Wnt binds to receptors Fzds and coreceptors LRPs, recruiting the destruction complex to the cell membrane via Dvl, which inhibits GSK‐3β from degrading β‐catenin. Extra β‐catenin in the cytoplasm translocates into the nucleus to promote targeting gene transcription. (B) The Wnt/PCP signaling pathway. Wnt ligands bind to the receptors Fzds and coreceptors Ror/Ryk, which activates downstream ROCK and JNK signaling via Dvl. (C) The Wnt/Ca^2+^ signaling pathway. Wnt‐Fzd binding leads to hydrolysis of PIP2 into DAG and IP3 to influence intracellular Ca^2+^ influx and regulate downstream gene expression. DAG, 1, 2‐diacylglycerol; Dvl, disheveled; Fzds, frizzleds; GSK‐3β, glycogen synthase kinase 3 protein; IP3, inositol 1, 4, 5‐triphosphate; JNK, c‐Jun N‐terminal kinases; LRPs, co‐receptor low‐density lipoproteins; PCP, planar cell polarity; PIP2, 4,5‐bisphosphate; ROCK, Rho‐associated protein kinases; Ror, tyrosine kinase‐like orphan receptor; Ryk, receptor tyrosine kinases. [Color figure can be viewed at wileyonlinelibrary.com]

The Wnt gene was originally derived from integrase‐1 in mouse breast cancer and the wingless gene of *Drosophila*.^63^ As shown in Figure 2, Wnt signaling pathways are typically classified into three branches depending on whether β‐catenin is involved: the canonical Wnt pathway (or Wnt/β‐catenin pathway) and the noncanonical Wnt pathway, including the Wnt planar cell polarity pathway (or Wnt/PCP pathway) and the Wnt/calcium pathway (or Wnt/Ca^2+^ pathway). The canonical Wnt pathway involves an accumulation of β‐catenin in the cytoplasm and its translocation into the nucleus to promote target gene transcription via T‐cell factor/lymphocyte enhancer factor (TCF/LEF) transcription factor. Without Wnt, β‐catenin is normally degraded by a destruction complex in the cytoplasm. The destruction complex consists of AXIN, adenomatous polyposis coli, casein kinase 1, glycogen synthase kinase 3 protein, and protein phosphatase 2A. This complex phosphates β‐catenin and targets it for degradation by the proteasome. On the other hand, when Wnt ligands are present, they bind to the transmembrane receptors Frizzled (Fzd) and co‐receptor low‐density lipoprotein (LRP), recruiting the destruction complex to the cell, which loses its ability to degrade β‐catenin (Figure 2A). The extra accumulation of β‐catenin in the cytoplasm leads to its translocation into the nucleus, where it activates the transcription of downstream genes by interacting with TCF/LEF.^64^, ^65^, ^66^ The noncanonical Wnt/PCP pathway is β‐catenin independent. As shown in Figure 2B, instead of using LRP, activation of the Wnt/PCP signaling is through using related to receptor tyrosine kinase, tyrosine kinase‐like orphan receptor 1/2, or tyrosine‐protein kinase‐like 7 as co‐receptor. Receptor binding leads to the internalization of the ligand‐receptor complex and downstream activation of Rho and Rac GTPases, rho‐kinase, and c‐Jun N‐terminal kinase, which together regulate cell motility and tissue polarity.^67^, ^68^ More recent studies have shown the important role of Wnt/Ca^2+^ signaling in cell development. The Wnt/Ca^2+^ pathway is initiated by Fzd receptors, activating a classical G protein‐coupled signaling pathway (Figure 2C). Fzd‐G protein signaling activates phospholipase C‐beta, which cleaves phosphatidylinositol 4,5‐bisphosphate into 1, 2‐diacylglycerol (DAG) and inositol 1, 4, 5‐triphosphate (IP3). Production of DAG and IP3 results in the activation of protein kinase C, calcium/calmodulin‐dependent protein kinase type II, and calcineurin phosphatase, which dephosphorylates and activates the transcription factor, nuclear factor associated with T cells. Genes activated through the Wnt/Ca^2+^ signaling regulate cell fate and cell migration.^68^, ^69^

### 3.2 Wnt signaling in regulating microglia

**Figure 3:**
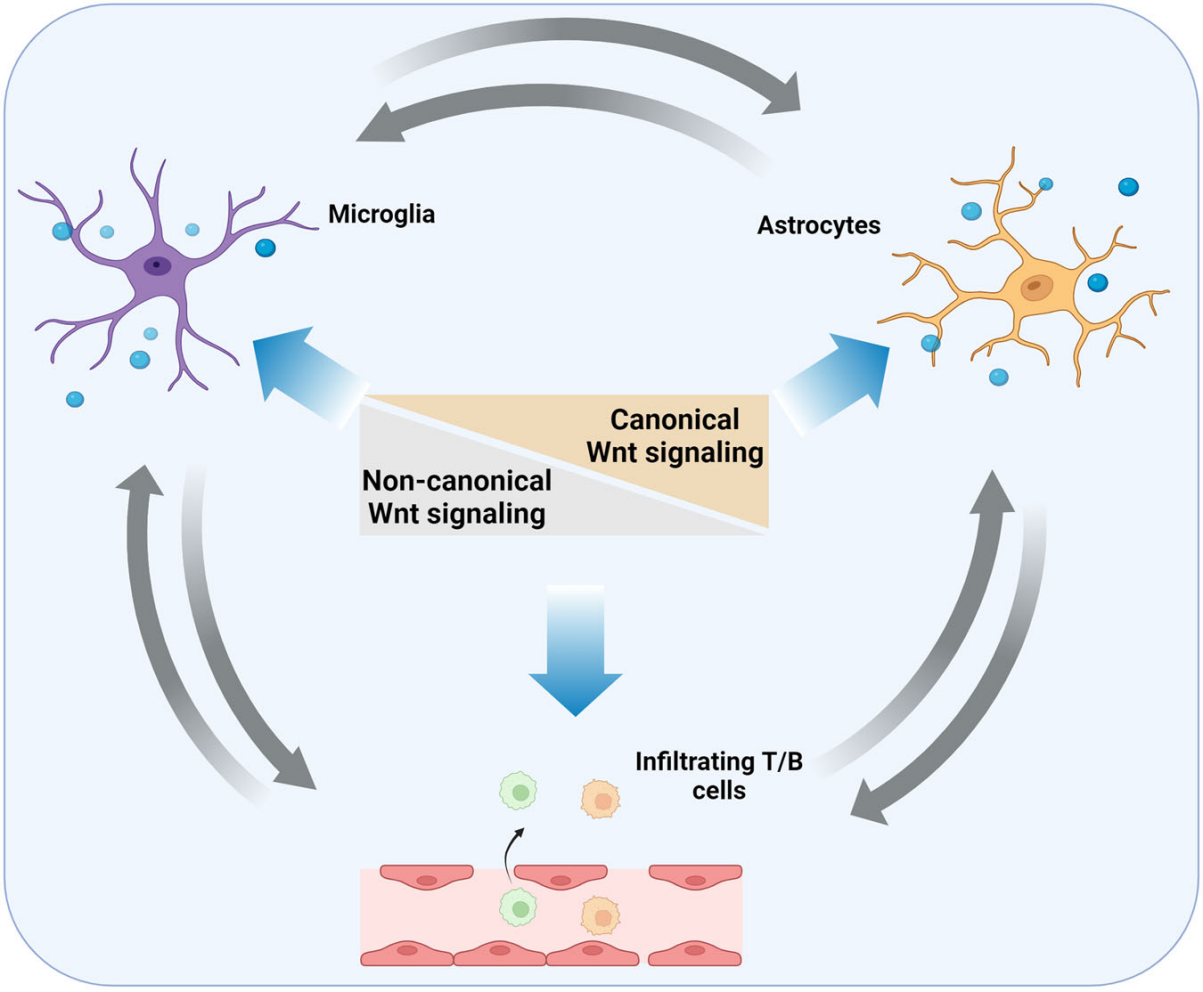
Wnt signaling in regulating immune response in the CNS. The canonical Wnt signaling and the noncanonical Wnt signaling antagonist each to keep balance in the CNS. This balanced Wnt signaling regulates the immune response via modulating the activation of microglia, astrocytes, and T/B cells. CNS, central nervous system. [Color figure can be viewed at wileyonlinelibrary.com]

Microglia, the resident immune cells of the CNS, are influenced by Wnt signaling (Figure 3). Several components of the Wnt pathway including Wnt ligands (e.g., Wnt1, Wnt3a, Wnt5a) and Fzd receptors (e.g., Fzd1, Fzd2, Fzd7) are expressed in microglia, and their activation or inhibition can markedly affect microglial function. For instance, Wnt3a inhibits the production of proinflammatory cytokines and enhances the expression of anti‐inflammatory factors. This shift toward an anti‐inflammatory state helps in reducing neuroinflammation and protecting neurons.^70^ By stabilizing β‐catenin and promoting its nuclear translocation, the canonical Wnt signaling can enhance the expression of genes in microglia such as BDNF and glial cell‐derived neurotrophic factor (GDNF) to support neuronal survival and synaptic plasticity. Conversely, dysregulation of Wnt signaling leads to an imbalance in microglial activation, contributing to chronic inflammation and neurodegeneration.^71^, ^72^ The noncanonical Wnt signaling pathways, including the Wnt/PCP pathway and the Wnt/Ca²⁺ pathway, are also involved in regulating microglial behavior. Through the Wnt/PCP pathway, Wnt5a influences microglial migration and phagocytosis, essential functions for clearing debris and damaged cells in the CNS. On the other hand, the Ca²⁺ influx induced by the Wnt/Ca²⁺ pathway modulates various aspects of microglial activation, including cytokine production and the oxidative stress response.^73^, ^74^

### 3.3 Wnt signaling in regulating astrocytes

Astrocytes, the resident immune cells in the CNS, are highly regulated by Wnt signaling (Figure 3). First, Wnt signaling is vital for the development and differentiation of astrocytes from neural progenitor cells. Additionally, this pathway is crucial for the maturation and functional specialization of astrocytes during development. In response to CNS injury or disease, astrocytes undergo reactive changes, known as astrogliosis. Wnt signaling, particularly the canonical pathway, modulates the extent and nature of this reactivity. Activation of the Wnt/β‐catenin signaling promotes a neuroprotective reactive astrocyte phenotype, which supports tissue repair and limits inflammation.^75^ Wnt signaling also influences the production of inflammatory mediators by astrocytes, thereby modulating the inflammatory environment in the CNS. Besides, Wnt signaling pathways regulate the release of gliotransmitters from astrocytes, influencing synaptic transmission and plasticity.^76^ The noncanonical Wnt/PCP pathway, activated by Wnt ligands like Wnt5a, regulates cytoskeletal organization, cell shape, and polarity, which are essential for astrocyte migration and the formation of the astrocytic network in the CNS.^76^, ^77^ The Wnt/Ca²⁺ pathway, involving intracellular calcium release, modulates astrocytic responses to injury and inflammation. For instance, Wnt5a‐mediated calcium signaling controls the production of cytokines and chemokines in astrocytes, participating in neuroinflammatory processes.^78^ Similarly, dysregulation of Wnt signaling leads to a detrimental astrocyte response, exacerbating neuroinflammation and tissue damage. For example, In AD, the disrupted Wnt/β‐catenin signaling in astrocytes is associated with increased neuroinflammation and impaired synaptic function.^79^

### 3.4 WNT signaling in regulating peripheral immune cells

As shown in Figure 3, Wnt signaling impacts the function and differentiation of peripheral immune cells, such as T cells and B cells. For example, in T cells, Wnt signaling helps progenitor cells differentiate into mature T cells within the thymus and influences the balance between different T cell subsets, such as regulatory T cells and effector T cells.^80^ In B cells, Wnt signaling impacts their maturation and antibody production.^81^ Wnt signaling is also important for the development and maintenance of the BBB through regulating the expression of tight junction proteins in endothelial cells, which are essential for maintaining the barrier function of the BBB.^81^ By controlling BBB integrity, Wnt signaling indirectly influences the trafficking of peripheral immune cells into the CNS, which is a key aspect of the immune response, particularly in the context of neuroinflammatory diseases.

## 4 WNT SIGNALING IN NEURODEGENERATIVE DISEASES

Wnt signaling is involved in many processes in the healthy CNS, from neuron development to homeostasis maintenance. However, dysregulated Wnt signaling is found to be linked with many neurodegenerative diseases, including AD, PD, and ALS.

### 4.1 Wnt signaling in AD

In the context of AD, Wnt signaling has garnered attention due to its role in neuroprotection, Aβ and tau pathology, and neuroinflammation. The canonical Wnt/β‐catenin pathway, in particular, inhibits the expression of β‐secretase (BACE1), an enzyme critical for the cleavage of amyloid precursor protein into amyloid‐β peptides.^82^, ^83^ In AD, reduced Wnt signaling leads to increased BACE1 activity, resulting in higher Aβ production and plaque formation. Moreover, Aβ accumulation and tau hyperphosphorylation are also linked to disrupted Wnt signaling. For instance, Aβ oligomers bind to Wnt receptors on the cell surface, such as Fzds, disrupting the canonical Wnt signaling. This suppression leads to decreased β‐catenin levels, impairing the transcription of neuroprotective genes (e.g., BDNF, NGF, GDNF).^84^ Deficiency in LDL receptor‐related protein 6 (LRP6) contributes to synaptic abnormalities and amyloid pathology in AD.^85^ Another study shows that Aβ directly interferes with disheveled segment polarity protein 1 (Dvl) to inhibit Wnt signaling in AD.^86^ And the abnormal accumulation of Aβ plaques leads to increased activity of glycogen synthase kinase 3 beta (GSK‐3β) resulting in decreased β‐catenin activity. On the other hand, high activation of GSK‐3β increases phosphorylated tau at multiple sites, promoting the formation of neurofibrillary tangles.^87^ Furthermore, Wnt signaling inhibitors, like dickkopf Wnt signaling pathway inhibitor 1 and dickkopf Wnt signaling pathway inhibitor 3, are overexpressed in the AD patient brains and they antagonist Wnt signaling by binding its co‐receptors LRP5/6.^88^, ^89^ In AD brains, Wnt signaling dysregulation is also related with chronic activation of microglia, which results in producing proinflammatory cytokines and neurotoxic substances.^90^ Activation of Wnt signaling has been suggested to offer neuroprotective effects in AD by promoting the survival of neurons and maintaining synaptic density. Enhancing Wnt/β‐catenin signaling could counteract Aβ toxicity and tau pathology, suggesting a potential therapeutic strategy for AD.

### 4.2 Wnt signaling in PD

Studies have underscored that many signaling pathways are disrupted in PD pathogenesis, with the Wnt signaling pathways emerging as a key player in this context. In PD, the neuroprotective role of Wnt1 and its receptor Frizzled‐1 (Fzd‐1) in dopaminergic neurons of the substantia nigra has been highlighted. However, abnormal activation of Wnt1/Fzd‐1 signaling leads to neurodegeneration in PD brains.^91^ Genetic mutations in Wnt signaling components also have been linked to PD, including LRP6, GSK‐3β, Dvl, and β‐catenin. For example, in the pathology of PD, GSK‐3β is often hyperactive, resulting in enhanced β‐catenin degradation and reduced Wnt signaling. Hyperactive GSK‐3β contributes to oxidative stress and apoptosis in dopaminergic neurons.^92^ Dvl mutations affect both canonical and noncanonical Wnt signaling. Altered Dvl function disrupt cytoskeletal dynamics and neuronal connectivity, which are critical in maintaining dopaminergic neuron integrity.^93^ Research indicates that a PD pathological mutation of LRRK2 gene leads to Wnt/β signaling deficit, demonstrating the role of Wnt signaling during PD pathogenesis.^94^ Neuroinflammation is a key component of PD pathology. Dysregulated Wnt signaling in the PD brains failed to regulate immune responses, resulting in proinflammatory effects, and causing neuronal damage.^95^ In addition to its role in neuronal survival and inflammation, Wnt signaling is vital for the synaptic plasticity of motor neurons. Activation of Wnt signaling can ameliorate cognitive deficits by restoring synaptic connection and enhancing plasticity in PD.^96^ One of the pathological hallmarks of PD is the accumulation of α‐synuclein aggregates in the form of Lewy bodies. Activation of Wnt/β‐catenin signaling was reported to reduce α‐synuclein aggregation and promote its clearance through the enhancement of autophagic pathways.^92^ All these studies suggest that the Wnt signaling pathway is integral to the pathogenesis and progression of PD, with genetic factors playing a significant role.

### 4.3 Wnt signaling in ALS

ALS is a progressive neurodegenerative disorder that affects nerve cells in the brain and spinal cord. Wnt signaling is involved in both neuroprotection and the pathological progress of ALS. One Wnt ligand from the Wnt/β‐catenin pathway, Wnt3a, has been shown to promote the survival of motor neurons in vitro, suggesting the protective function of the Wnt/β‐catenin signaling in ALS brains.^97^ However, during the development of ALS, decreased expression of Wnt3a and β‐catenin was observed in spinal cord and motor neurons.^98^, ^99^ SOD1 mutations are among the most common genetic causes of familial ALS. Studies have shown that SOD1 mutations disrupt Wnt signaling, leading to impaired neuronal survival and increased susceptibility to oxidative stress.^99^ Another hallmark of ALS is abnormal aggregation of TDP‐43. Research indicates that TDP‐43 can modulate the expression of Wnt pathway genes and aberrant TDP‐43 function leads to dysregulation of Wnt signaling, contributing to neuronal vulnerability.^98^, ^100^ Secreted frizzled‐related protein, an inhibitor of Wnt signaling, was found to be upregulated in the ALS brains, contributing to motor neuron degeneration.^98^ Also, disrupted Wnt signaling has failed to regulate the activation and function of glial cells, which are crucial in the neuroinflammatory response in ALS. For example, in the ALS mice models, enhancing Wnt signaling in microglia was associated with reduced neuroinflammation and motor neuron preservation.^101^ All these studies improve the critical regulator of Wnt signaling in the pathogenesis of ALS.

Common cell signaling pathways, mainly phosphatidylinositol 3‐kinase/protein kinase B NF‐κB, Notch, and TGF‐β are also involved in neurodegenerative diseases (Table 1). These pathways crosstalk with Wnt signaling pathways to contribute to neurogenesis, neural survival, and immune responses in the healthy CNS. However, during the development of neurodegenerative diseases, dysregulation of these signaling pathways accelerates negative effects of Wnt signaling, such as synaptic loss, oxidative stress, and neuroinflammation. As the central modulator of cell signaling, Wnt signaling represents a potential therapeutic target across these neurodegenerative diseases. Understanding and targeting the nuances of Wnt signaling could pave the way for more effective interventions in these devastating disorders.

**Table 1:** Crosstalk between common cell signaling and Wnt signaling pathways in neurodegenerative diseases.

Signaling pathway	Role in neurodegenerative disease	Interaction with Wnt signaling	References
Wnt signaling	– Regulates neurogenesis, synaptic function, and plasticity – Dysregulation leads to synaptic loss and neurodegeneration		[102]
PI3K/AKT signaling	– Modulates glucose metabolism and energy production to promote cell survival – Dysregulation accelerates oxidative stress and apoptosis of neurons	– PI3K/AKT enhances β‐catenin stabilization to promote cell survival and proliferation – Disrupted Wnt/PI3K/Akt axis leads to increased apoptosis and reduced neuronal survival	[103, 104]
NF‐κB signaling	– Regulates the expression of proinflammatory cytokines to protect neurons – Chronic activation leads to neuroinflammation and neuronal damage	– Wnt modulates NF‐κB activity to reduce neuroinflammation – Chronic activation of NF‐κB inhibits Wnt signaling to reduce neuroprotective effects	[105, 106]
Notch signaling	– Controls the fate of neural stem cells and the formation of neurons – Dysregulation impairs neurogenesis and synaptic function	– Notch and Wnt antagonist each other to determine cell fate – Imbalanced between Notch and Wnt signaling induces neural death	[107, 108]
TGF‐β signaling	– Controls inflammation and extracellular matrix production – Dysregulation results in increased neuroinflammation and neurodegeneration	– TGF‐β synergizes with Wnt to promote cell survival – Dysregulation of Wnt/TGF‐β exacerbates neuroinflammation and neurodegeneration	[109, 110]

Abbreviations: AKT, protein kinase B; NF‐κB, nuclear factor‐kappaB; PI3K, phosphatidylinositol 3‐kinase; TGF‐β, transforming growth factor beta.

## 5 DRUG DEVELOPMENT TARGETING WNT SIGNALING AND NEUROINFLAMMATION FOR NEURODEGENERATIVE DISEASES

The development of drugs targeting Wnt signaling pathways and neuroinflammation represents a promising avenue for the treatment of neurodegenerative diseases. These conditions are characterized by chronic neuroinflammation and progressive neuronal loss, which are often exacerbated by dysregulated Wnt signaling. By modulating Wnt signaling, it is possible to influence immune responses, reduce inflammation, and promote neuroprotection, offering potential therapeutic benefits. One of the primary strategies in drug development targeting Wnt signaling involves the use of small molecules or biologics that can either activate or inhibit specific components of the Wnt pathways. For instance, treatment of cultured microglia with Wnt3a reduces proinflammatory cytokine production and increases anti‐inflammatory cytokine levels.^111^ Inhibitors of GSK‐3β have shown promise in preclinical studies by promoting Wnt pathway activity, reducing inflammation, and protecting neurons from degeneration.^112^ Similarly, small molecules that enhance the stability and activity of β‐catenin are being investigated for their potential to ameliorate symptoms and slow the progression of neurodegenerative diseases.^113^ In addition to direct modulation of Wnt signaling, targeting the interaction between Wnt signaling and neuroinflammatory processes is another therapeutic strategy. Compounds that inhibit proinflammatory cytokines or block the activation of microglia and astrocytes, thereby reducing neuroinflammation, are being explored. For example, nonsteroidal anti‐inflammatory drugs and other anti‐inflammatory agents that modulate the Wnt pathway have demonstrated neuroprotective effects in animal models of neurodegenerative diseases.^114^

## 6 FUTURE PERSPECTIVES

Drug development targeting Wnt signaling and neuroinflammation offers a multifaceted approach to treating neurodegenerative diseases. The Wnt signaling pathway encompasses multiple components, including ligands, receptors, co‐receptors, and intracellular signaling molecules. This provides a broad spectrum of potential therapeutic targets for drug development. Future research should focus on unraveling the precise mechanisms by which Wnt signaling interacts with immune cells in the CNS. This includes understanding the context‐dependent effects of Wnt signaling on microglia, astrocytes, and the BBB across different stages of neurodegenerative diseases. Cross‐disciplinary technology provides new ideas for clinical therapy of neurodegenerative diseases. For example, the pathology of AD has been increasingly explored through single‐cell and single‐nucleus RNA‐sequencing and spatial transcriptomics.^115^ The application of computational science in the biological field has greatly accelerated research in targeted drug development. Utilizing these advanced technologies can help delineate the specific roles of Wnt signaling in various cell types and subtypes within the CNS and uncover the heterogeneity of cellular responses to Wnt modulation, providing targeted insights.

However, there are limitations of Wnt‐targeting drug development in immune response in neurodegenerative diseases. For example, highly selective Wnt pathway modulators that can specifically target canonical or noncanonical pathways are still deficient. Therapeutic modulation of Wnt signaling must be approached with caution due to its widespread involvement in various physiological processes, including bone formation and tissue regeneration. Achieving specificity in targeting Wnt pathways is critical to avoid potential side effects and ensure the efficacy of treatments. Another challenge is the efficient delivery of drugs into the CNS. The presence of the BBB in the CNS complicates drug delivery, ensuring adequate drug penetration while maintaining BBB integrity is a significant issue. New techniques, like the Adeno‐associated virus delivery system, are worth the researcher's attention for their potential to deliver drugs efficiently into the CNS. Furthermore, variability in genetic and epigenetic factors among patients can influence the effectiveness of Wnt‐targeting therapies. Moreover, personalized approaches may be required, complicating the development and application of these treatments. Treatment for neurodegenerative diseases is a lifelong process study. Continued research and clinical trials are essential to fully realize the therapeutic potential of Wnt‐targeted interventions in neurodegenerative diseases.

## 7 CONCLUSION

In conclusion, Wnt signaling pathways play a vital role in regulating immune responses in the CNS, which are essential for both neuroprotective and neurodegenerative effects. By modulating the activity of various immune cells, such as microglia and astrocytes, Wnt signaling helps maintain a delicate balance between protective and harmful immune responses in the CNS. In the neuroprotective scenarios, Wnt signaling promotes the survival and function of neurons by enhancing the anti‐inflammatory properties of glial cells and reducing the production of proinflammatory cytokines. This balance is crucial in preventing the onset and progression of neurodegenerative diseases, where excessive inflammation and immune dysregulation lead to neuronal damage and loss. Additionally, Wnt signaling facilitates the repair and regeneration of neural tissues by influencing the proliferation and differentiation of neural progenitor cells, further underscoring its importance in maintaining neural health.

Apart from the neuroprotective effect of Wnt signaling, dysregulation of this signaling has been implicated in various neurodegenerative diseases. In these conditions, aberrant Wnt signaling contributes to chronic neuroinflammation, exacerbating disease pathology and progression. In AD, for instance, impaired Wnt signaling is associated with increased Aβ production and tau hyperphosphorylation, leading to neurotoxicity and cognitive decline. Similarly, in PD, disrupted Wnt signaling leads to dopaminergic neuron loss and motor dysfunction. Understanding the mechanisms by which Wnt signaling influences immune responses offers potential therapeutic avenues for modulating immune activity to protect neurons and reduce inflammation. Targeting specific components of the Wnt pathway could provide novel strategies to restore immune balance, enhance neuroprotection, and ultimately improve outcomes in patients with neurodegenerative diseases. Therefore, continued research into the Wnt signaling pathway and its interactions with the immune system holds promise for the development of effective treatments for these debilitating conditions.

## AUTHOR CONTRIBUTIONS

Kevin Fang conceived, designed, and wrote the manuscript.

## CONFLICT OF INTEREST STATEMENT

The author declares no conflict of interest.

## ETHICS STATEMENT

Not applicable.

## Data Availability

No new data were generated in this review. All the data cited in this study are available from the original publications.
